# Intergenerational transmission of parenting: findings from a UK longitudinal study

**DOI:** 10.1093/eurpub/ckv093

**Published:** 2015-06-02

**Authors:** Vaishnavee Madden, Jill Domoney, Katie Aumayer, Vaheshta Sethna, Jane Iles, Isabelle Hubbard, Andreas Giannakakis, Lamprini Psychogiou, Paul Ramchandani

**Affiliations:** 1 Department of Primary Care and Public Health, Imperial College, London, UK; 2 Imperial College, The Centre for Mental Health, London, UK; 3 Institute of Psychiatry, Psychology and Neuroscience, King's College London, London, UK; 4 Academic Unit of Child and Adolescent Psychiatry, Imperial College London, London, UK; 5 Department of Psychology, University of Bath, Bath, UK; 6 Department of Psychiatry, University of Oxford, Oxford, UK; 7 Department of Psychology, University of Exeter, Exeter, UK

## Abstract

**Background: **The quality of parenting is associated with a wide range of child and adult outcomes, and there is evidence to suggest that some aspects of parenting show patterns of intergenerational transmission. This study aimed to determine whether such intergenerational transmission occurs in mothers and fathers in a UK birth cohort. **Methods: **The study sample consisted of 146 mothers and 146 fathers who were recruited from maternity wards in England and followed up for 24 months [‘Generation 2’ (G2)]. Perceptions of their own parenting [by ‘Generation1’ (G1)] were assessed from G2 parents at 12 months using the Parental Bonding Instrument (PBI). G2 parents were filmed interacting with their ‘Generation 3’ (G3) children at 24 months. **Results: **We found that G1 mothers’ ‘affection’ was associated with positive parenting behaviour in the G2 fathers (‘positive responsiveness’ *β* = 0.19, *P* = 0.04 and ‘cognitive stimulation’ *β* = 0.26, *P* < 0.01). G1 mothers’ ‘control’ was associated with negative parenting behaviour in G2 mothers (decreased ‘engagement’ *β* = −0.19, *P* = 0.04), and negative parenting behaviour in G2 fathers (increased ‘control’ *β* = 0.18, *P* = 0.05). None of the G1 fathers’ parenting variables were significantly associated with G2 parenting. **Conclusions: **There is evidence of intergenerational transmission of parenting behaviour in this highly educated UK cohort, with reported parenting of grandmothers associated with observed parenting in both mothers and fathers. No association was seen with reported parenting of grandfathers. This raises the possibility that parenting interventions may have benefits that are realised across generations.

## Background

It is well established that the quality of parenting is associated with a wide range of child outcomes, including adjustment,^1^ emotional and behavioural problems^2^ and physical health,^3^ as well as subsequent mental health problems later in life.^4^ Warm and supportive parenting is associated with academic achievement, psychosocial development and emotional stability.^5^ Conversely, harsh parenting is associated with child aggression and conduct problems.^6^ As such, it is important to understand the factors that influence these parenting behaviours so that these factors may be targeted in interventions.

One important influence on parenting is its intergenerational transmission: that is, the influence of parents’ own experiences as a child on their later childrearing practices.^7^ There is a body of evidence to suggest such transmission exists, although only to a mild to moderate degree, with estimates suggesting an average of 35–45% of parenting behaviour transmitted to the next generation.^8^ The majority of studies have focused on the intergenerational continuity of harsh, aggressive parenting, but more recent studies have confirmed that this is also true for warm, supportive parenting.^9–12^

Intergenerational transmission of parenting can be explained by direct mechanisms. For example a child observes his/her parent’s behaviour, and emulates this parental style when becoming a parent (‘Social Learning’ theory)^13^ or a child develops an attachment style as a result of parent–infant interaction, which is replicated when the child becomes a parent.^14^ Such continuity in parenting behaviour may also be indirect, and mediated by some other intermediate factor. For example, child and adolescent antisocial behaviour (extending into adulthood) has been proposed to mediate the transmission of harsh/aggressive parenting^15^ Similarly, the development of the child into a competent adult (measured by educational attainment or positive peer relations) has been proposed to mediate the transmission of warm, supportive parenting.^12^^,^^16^^,^^17^ In addition to all of these putative ‘social-environmental’ mechanisms to explain intergenerational transmission of parenting, lies the possibility that there is a genetic component to some aspects of parenting behaviour.^9^

Intergenerational transmission of parenting has been replicated by studies set in different countries and in different socio-demographic samples.^9^ However previous studies have mainly used self-reported measures of parenting, potentially subject to reporting biases, with only a few having used more objective measures of observed parenting. Additionally many focus only on mothers, rather than both parents. Furthermore, there has only been one other UK study investigating this issue, which focused on how a history of childhood abuse impacted on parenting behaviour.^18^ In this current study, we aim to add to the evidence base by testing the hypothesis that there is intergenerational transmission of parenting in a UK sample, using an observed measure of parenting, and to assess the transmission of both positive and negative parenting behaviours in both mothers and fathers. Assessing the possibility of intergenerational transmission requires studying at least three generations: generation 1 (G1), generation 2 (G2) and generation 3 (G3).^7^ We specifically aim to determine the influence of the parenting received from G1 mothers and G1 fathers on the parenting displayed by G2 mothers and G2 fathers.

## Methods

### Participants

The Oxford Fathers Study is a longitudinal cohort study of 192 couples followed up for 2 years from the birth of their child. Participants were recruited from postnatal maternity wards of hospitals in Oxford and Milton Keynes, England. The aim of the study was to examine the early influence of fathers on their children’s development, with a particular focus on paternal mood, and recruitment aimed to oversample fathers with depression. The study was approved by the Oxfordshire Research Ethics Committee.

The couples were subsequently contacted and visited at home at 3 time points (3 months, 12 months and 24 months). Of the 192 families initially recruited, 24 month parent–child interaction data was available for 147 families. Of these 147 families, 1 family was excluded due to missing data (Parental Bonding Instrument: PBI) at the 12 month home visit. As such, the current study population comprised 146 mothers and 146 fathers.

### Definition of the generations

G2 mothers and G2 fathers were the recruited couples. G1 mothers and G1 fathers were the parents of G2 mothers or G2 fathers. G3 infants were the children of G2 couples.

**Figure 1:**
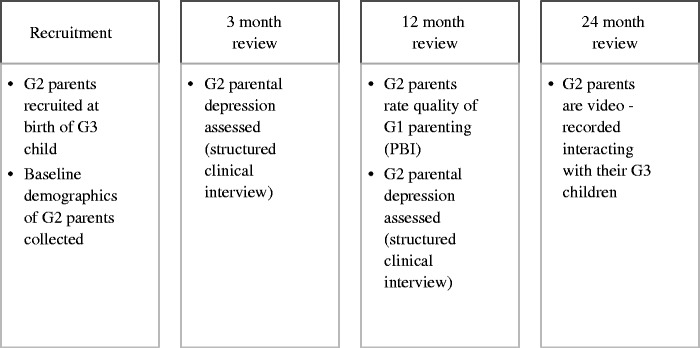
Key study milestones and measures.

Figure 1 demonstrates the key study milestones, in terms of participants and measures.

## Measures

### Parental bonding instrument (G1 parenting)

Quality of G1 parenting was based on questionnaires administered to G2 mothers and G2 fathers during home visits by members of the research team at 12 months. The Parental Bonding Instrument (PBI), which was developed to enable systematic investigation of the effect of parenting on psychopathology, is a 25-item self reported measure, with subjects scoring their parents’ attitudes and behaviours as remembered for their first 16 years.^19^ It measures two theoretically and empirically derived dimensions of parental bonding: parental affection and parental control. High scores of parental affection refer to being emotionally available, attentive and interested in the child. High scores on parental control refer to being manipulative, arbitrary or harsh in disciplining the child.^19^ ‘Affectionless control’ (low affection and high control) has been demonstrated to be a risk factor for psychopathology in later life, including antisocial personality traits,^20^ depression,^21^ and anxiety disorders.^22^ The PBI has been shown to have good construct and convergent validity,^19^ as well as reliability in both the short and long term.^23^

### Parent–child interactions at 24 months (G2 parenting)

Quality of G2 parenting was assessed by video-recorded observations of parent–child interaction at the 24 month home visit. G2 mothers and G2 fathers were separately filmed interacting with their G3 child on a floor mat for 2 minutes without any toys (‘free play’) and then with a book (‘book session’) for 5 minutes. These interactions were coded using a scheme designed to take into account the range of behaviours seen in parent child interactions with 2 year olds and to account for some of the different behaviours that fathers have been noted to display (e.g. physical interaction).^24^ This coding scheme was based on the Global Rating Scales (GRS), a video-based assessment of the quality of mother–infant interactions,^25^ and further developed. Parental behaviour was initially rated on 20 dimensions (sensitivity, following child’s attention, withdrawal, intrusions, educational references, elaboration, strong control, facilitating child’s attention, positive expressed emotion, negative expressed emotion, warmth, imitation of the child, direct emotion/warm touching, emotional tone, anxiety, self-referential/helplessness, parental attention, physical interaction during play, instrumental touching, acknowledgement of child as a separate agent). Parent–child interaction was rated on 2 dimensions (conflictual behaviour and reciprocity/synchronicity). The videotaped interactions were scored by trained researchers who had not been involved in the home visit, and so were blind to family characteristics. There was moderate to good inter-rater agreement on each dimension, with the average weighted kappa ranging from 0.56 to 0.69.

To reduce the number of variables for analysis, the variables were subject to data reduction, including principle components analysis. For G2 mothers, 2 factors emerged from the free play session (‘positive responsiveness’ and ‘engagement’), which explained 50% of the variance, and 3 factors from the book session (‘positive responsiveness’, ‘cognitive stimulation’ and ‘control’), which explained 60% of the variance. For G2 fathers, 3 factors emerged from the free play session (‘positive responsiveness’, ‘negative responsiveness’ and ‘engagement’), which explained 66% of the variance, and 3 factors from the book session (‘positive responsiveness’, ‘control’ and ‘cognitive stimulation’), which explained 52% of the variance. High scores on each of these factors represented higher levels of each behaviour. Further details of the factor analysis is described elsewhere.^26^

### Covariates

The following socio-demographic variables were included as covariates in the analyses:

1. Parental age, as research has demonstrated that experiencing adverse parenting in childhood is associated with early onset of parenthood. ^27^
2. Socio-economic status, since adverse parenting in both generations can be associated with socioeconomic disadvantage.^28^^,^^29^ In this study, parental household social class was measured by the occupational social class of the fathers, using the groupings of the UK Office for National Statistics Socio-Economic Classifications, and coded into four categories: professional/managerial, intermediate, routine/manual and unemployed.
3. Parental depression, as in addition to being a potential consequence of adverse parenting,^21^ depression may also affect displayed parenting and colour the recollection of the parenting received.^30^ Maternal and paternal depression were assessed at the 3 month and 12 month home visits by a Structured Clinical Interview for Depression (SCID). Postnatal depression (at either 3 months or 12 months), as diagnosed using Diagnostic and Statistical Manual of Mental Disorders, 4th Edition (DSM IV) criteria, was coded as either ‘present’ or ‘absent’ for G2 mothers and G2 fathers.

### Statistical analysis

All analyses were conducted on Statistical Package for the Social Sciences (SPSS) version 20 and all tests were two tailed. First, sample characteristics were described, and presented with proportions for categorical variables, and means and standard deviations for continuous variables. Second, bivariate correlations were conducted to examine the associations between predictor (Parental Bonding Instrument scores) and outcome (parent–child observed interaction at age 2 years) variables. Where associations were found, multiple linear regression analyses were conducted to control for the effect of potential confounding variables.

## Results

The baseline socio-demographic characteristics of the sample are shown in table 1. The mean age was 33.21 years for mothers and 34.74 years for fathers. The majority of parents were white (93% of mothers and 95% of fathers), highly educated (62% of mothers and 62% of fathers had a degree or postgraduate qualification) and were employed in professional/managerial occupations (56% of families).

**Table 1:** Descriptive characteristics of G2 parents

Variable	Mothers	Fathers
	Mean (sd)	Mean (sd)
Age (when index child born)	33.2 (4.7)	34.7 (5.7)
Parental education:		
No qualifications	–	1 (0.7%)
GCSE	8 (5.5%)	13 (8.9%)
A levels or equivalent	14 (9.6%)	13 (8.9%)
Diploma or equivalent degree	27 (18.5%)	25 (17.1%)
Degree	46 (31.5%)	51 (34.9%)
Postgraduate	46 (31.5%)	40 (27.4%)
Missing	5 (3.4%)	3 (2.0%)
Parental occupation:	
Managerial/professional	81 (55.5%)
Intermediate occupations	39 (26.7%)
Routine/manual	25 (17.1%)
Unemployed	1 (0.7%)

Tables 2 and 3 show the Pearson correlation coefficients between G1 parenting and aspects of G2 parenting for mothers (table 2) and fathers (table 3). Table 2 shows that G1 mothers’ ‘control’ is negatively associated with G2 mothers’ ‘engagement’ (correlation coefficient = −0.20, *P* = 0.03). Table 3 shows that G1 mothers’ ‘affection’ is positively associated with both G2 fathers’ ‘positive responsiveness’ (correlation coefficient = 0.21, *P* = 0.02) and G2 fathers’ ‘cognitive stimulation’ (correlation coefficient =0.28, *P* < 0.01). Table 3 also shows that G1 mothers’ ‘control’ is positively associated with G2 fathers’ ‘control’ (correlation coefficient=0.18, *P* = 0.05). Although most of the correlations were relatively small in magnitude, the statistically significant associations were all in the expected direction.

**Table 2:** Bivariate Pearson correlation coefficients (and significance levels) for G1 parenting measures and G2 mothers’ displayed parenting

Variables	‘Positive responsiveness’ (free play)	‘Engagement’ (free play)	‘Positive Responsiveness’ (book session)	‘Cognitive Stimulation’ (book session)	‘Control’ (book session)
**Predictors**					
G1 mother affection	−0.08 (0.41)	−0.03 (0.76)	−0.04 (0.66)	−0.07 (0.44)	−0.13 (0.15)
G1 mother control	−0.06 (0.51)	−0.20 (0.03)	−0.07 (0.47)	−0.09 (0.31)	0.16 (0.07)
G2 father affection	−0.10 (0.29)	−0.09 (0.35)	−0.09 (0.35)	0.00 (1.00)	0.15 (0.11)
G1 father control	0.01 (0.95)	−0.03 (0.78)	0.04 (0.67)	−0.02 (0.86)	−0.05 (0.61)

**Table 3:** Bivariate Pearson correlation coefficients (and significance levels) for G1 parenting measures and G2 fathers’ displayed parenting

Variables	‘Positive responsiveness’ (free play)	‘Negative responsiveness’ (free play)	‘Engagement’ (free play)	‘Positive Responsiveness’ (book session)	‘Control’ (book session)	‘Cognitive Stimulation’ (book session)
*Predictors*						
G1 mother affection	0.21 (0.02)	0.13 (0.17)	−0.01 (0.93)	0.12 (0.20)	−0.03 (0.71)	0.28 (0.002)
G1mother control	−0.05 (0.59)	−0.04 (0.65)	−0.02 (0.87)	0.03 (0.77)	0.18 (0.05)	−0.06 (0.54)
G1 father affection	0.17 (0.07)	−0.09 (0.35)	0.09 (0.36)	0.07 (0.43)	0.00 (1.00)	0.06 (0.49)
G1 father control	−0.06 (0.53)	0.02 (0.86)	−0.06 (0.49)	0.05 (0.58)	0.16 (0.09)	0.08 (0.39)

**Table 4:** Summary of multiple regression analyses, controlling for G2 parental age, socioeconomic status and depression

G1 parental variable	Factor	Unadjusted *β* coefficient	*P*-value	Adjusted *β* coefficient	*P*-value
G1 maternal affection	G2 father ‘positive responsiveness’	0.21	0.02	0.19	0.04
	G2 father ‘cognitive stimulation’	0.28	0.002	0.26	0.005
G1 maternal control	G2 mother ‘engagement’	−0.20	0.03	−0.19	0.04
	G2 father ‘control’	0.18	0.05	0.18	0.05

After controlling for the effects of potential confounding variables, G1 mothers’ ‘affection’ was still associated with G2 fathers’ ‘positive responsiveness’ (standardised *β* = 0.19, *P* = 0.04) and ‘cognitive stimulation’ (standardised β = 0.26, P < 0.01). G1 mothers’ ‘control’ was also still negatively associated with G2 mothers’ ‘engagement’ (standardised *β* = −0.19, *P* = 0.04). However, there was only weaker evidence for an association between G1 mothers’ ‘control’ and G2 fathers’ ‘control’ (standardised *β* = 0.18, *P* = 0.05).

## Discussion

### Main findings of this study

This study adds to the body of evidence demonstrating that there is some intergenerational transmission of parenting characteristics in both mothers and fathers in a UK sample. We found that a higher level of affection by grandmothers is associated with more positive parenting behaviour in fathers (more ‘positive responsiveness’ and more ‘cognitive stimulation’). A higher level of control by grandmothers is associated with lower engagement by mothers. None of grandfathers’ parenting variables are associated with the parenting behaviour of mothers and fathers.

### Strengths and limitations

This study overcomes some of the biases of previous studies, for example the common method variance bias in studies that rely on a single G2 informant to assess both G1 and G2 parenting.^31^ The use of videotaped interactions of G2 parent with G3 child, when rated blind, as in this study, is considered to be a more objective measure,^32^ and has only been used in a small number of studies investigating intergenerational transmission of parenting. Another strength is the longitudinal design, with measures of G1 parenting and G2 observed parenting assessed at different times. Furthermore, the use of a non-clinical sample may reduce some of the selection bias associated with clinical populations.

There are also several limitations of this study, including limitations of the measures used. Although the measure of G1 parenting—the PBI—is an extensively used, reliable and valid measure, it is still a retrospective one, with the possibility of recall bias. Measurement error is also likely to have occurred for the measures of parenting (for example, the inter-rater reliability of the GRS scale was around 0.6). With two imprecise measures, the chances of finding high correlations even when the underlying constructs are highly correlated are reduced. Furthermore, controlling for socio-demographic and parental depression, may reduce the size of any effect because both can be outcomes of G1 parenting in addition to being correlated with G2 parenting. This element of over-control may once again increase the chance of a type 2 error for this study.

The measures used for this study are measuring non-identical constructs of parenting, with the PBI measuring recall of parenting in childhood in general (first 16 years) and the videotaped observations of G2 parent-G3 child interaction measuring observed parenting at a single developmental period (24 months). Since parenting behaviour is a complex phenomenon with many potential determinants, there is also possibility of unknown or unmeasured confounding variables, not accounted for in our model.

It should be noted that the G2 sample was fairly homogeneous (white, higher than average socio-economic status), with consequent limitations to the generalisability of the findings. However, this is mitigated by the fact that several other studies have demonstrated intergenerational transmission of parenting in samples from lower socio-economic groups and from different cultural backgrounds.^9^

Although the study results, demonstrating modest intergenerational transmission of certain parenting characteristics, should be considered with caution given the lack of associations observed for many of the other parenting outcome variables in the bivariate correlation analyses, this should also be interpreted in the context of the likelihood of type 2 error.

### Comparison with other studies

This study adds to the evidence base regarding intergenerational transmission of parenting in several ways. First, it confirms previous findings about the intergenerational transmission of parenting. The associations found in this study (effect size 0.18–0.28) are similar in magnitude to previous studies (around 0.20–0.40),^9^ confirming the robustness of findings across diverse study samples and with different types of measurement. There has been only one other UK study to address this issue, which focused only on the transmission of negative parenting behaviours, and demonstrated that parents with a history of abuse were more likely to display poor quality parenting behaviour.^18^ Such studies of families with a history of abuse do not necessarily generalise to the wider parenting population. The majority of the extant literature on the intergenerational transmission of parenting, including the other UK study, has focused on abusive or harsh parenting, with a smaller number of studies investigating the continuity of warm or supportive parenting.^10^^,^^11^ This study is one of the few studies to assess the intergenerational transmission of both positive and negative parenting behaviours, and as such serves to extend the evidence base.

Second, this is also one of the few studies to investigate separately the parenting behaviours of mothers and fathers. The finding that the parenting behaviour of mothers and fathers is associated with grandmothers’ parenting and not grandfathers’, is an interesting one, and potentially highlights a greater role that the mother played as a primary caregiver, with a greater influence on their child’s ‘learned’ parenting^13^ or attachment style.^14^ It should be noted that time trends in parental involvement in their children’s lives, with increased paternal involvement in many families in recent years, may mean that this finding may not be as relevant today as it was in the grandparenting generation. The finding that grandmothers’ parenting exerted a greater influence on the parenting behaviour of fathers, may be due to the greater number of alternative influences on the mothers’ parenting behaviour compared to the fathers (e.g. time spent with child, other forms of social support, etc). The impact of gender on parenting behaviour has produced mixed findings. While some studies have demonstrated that intergenerational transmission of parenting behaviour occurs for daughters and not for sons,^10^ other studies have found no such differences.^9^

### Implications and future direction

Recent UK public health policy has embraced both a life-course and social determinants perspective.^33^ Parenting plays a fundamental role in child development, which is central to health, social and educational outcomes in later life.^1–3^ As such, it is of utmost importance to society that we have a greater understanding of the complex issue of parenting behaviour. Furthermore, clinicians and commissioners of parenting interventions should be aware that the benefits of parenting interventions are likely to be realised in both the short term and in the long term, across generations. Most existing health economic studies of parenting interventions only consider short-term benefits, and as such, are likely to underestimate the longer term benefits because such parenting interventions could also interrupt inter-generational transmission. The fact that there are potential gains to be made in parenting even in highly educated families may support the case for universal parenting interventions.

The modest correlations demonstrating some intergenerational transmission may be due to discontinuity in parenting behaviour, but may also be due in part to measurement error.^34^ The evidence for the influence of moderating factors that may explain such discontinuity is sparse, but may include the role of gender,^10^^,^^35^ relationship quality^11^^,^^36^ and educational attainment.^17^^,^^37^ Further studies are required to find out why some people repeat the parenting behaviours they experienced while growing up, yet others do not. Elucidating the mediating factors and mechanisms that contribute to the continuity of positive parenting behaviours and moderating factors that contribute to discontinuity of negative parenting behaviours will be crucial avenues of future research. Better understanding of the processes involved in the complex phenomenon of human parenting, will be useful to inform interventions aimed at breaking intergenerational cycles of poor parenting practices^38^ and to inform policy.

## Conclusion

There is evidence of intergenerational transmission of both positive and negative parenting behaviour in this highly educated UK cohort, with grandmothers’ reported parenting associated with observed parenting in both mothers and fathers. This raises the possibility that parenting interventions may have benefits that are realised across generations.

## Funding

This research was supported by a Wellcome Trust Clinical Research Fellowship (078434) to P.G.R. and a NIHR Academic Clinical Fellowship to V.M.

Key points

- Parenting is a well-established determinant of child and adult health and social outcomes
- This study suggests that key aspects of parenting in both mothers and fathers are influenced by their own parenting experiences (intergenerational transmission of parenting)
- This raises the possibility that parenting interventions may have benefits that are realised across generations
